# Frequency of rare mutations and common genetic variations in severe hypertriglyceridemia in the general population of Spain

**DOI:** 10.1186/s12944-016-0251-2

**Published:** 2016-04-23

**Authors:** Itziar Lamiquiz-Moneo, Cristian Blanco-Torrecilla, Ana M. Bea, Rocío Mateo-Gallego, Sofía Pérez-Calahorra, Lucía Baila-Rueda, Ana Cenarro, Fernando Civeira, Isabel de Castro-Orós

**Affiliations:** Unidad Clínica y de Investigación en Lípidos y Arteriosclerosis, Hospital Universitario Miguel Servet, Instituto de Investigación Sanitaria Aragón (IIS Aragón), Avenida Isabel La Católica 1-3, 50009 Zaragoza, Spain; Universidad de Zaragoza, Departamento de Bioquímica, Biología Molecular y Celular, 50009 Zaragoza, Spain

**Keywords:** Hypertriglyceridemia, Mutations, Prevalence

## Abstract

**Background:**

Hypertriglyceridemia (HTG) is a common complex metabolic trait that results of the accumulation of relatively common genetic variants in combination with other modifier genes and environmental factors resulting in increased plasma triglyceride (TG) levels. The majority of severe primary hypertriglyceridemias is diagnosed in adulthood and their molecular bases have not been fully defined yet. The prevalence of HTG is highly variable among populations, possibly caused by differences in environmental factors and genetic background. However, the prevalence of very high TG and the frequency of rare mutations causing HTG in a whole non-selected population have not been previously studied.

**Methods:**

The total of 23,310 subjects over 18 years from a primary care-district in a middle-class area of Zaragoza (Spain) with TG >500 mg/dL were selected to establish HTG prevalence. Those affected of primary HTG were considered for further genetic analisys. The promoters, coding regions and exon-intron boundaries of *LPL*, *LMF1*, *APOC2*, *APOA5*, *APOE* and *GPIHBP1* genes were sequenced. The frequency of rare variants identified was studied in 90 controls.

**Results:**

One hundred ninety-four subjects (1.04 %) had HTG and 90 subjects (46.4 %) met the inclusion criteria for primary HTG. In this subgroup, nine patients (12.3 %) were carriers of 7 rare variants in *LPL*, *LMF1, APOA5, GPIHBP1* or *APOE* genes. Three of these mutations are described for the first time in this work. The presence of a rare pathogenic mutation did not confer a differential phenotype or a higher family history of HTG.

**Conclusion:**

The prevalence of rare mutations in candidate genes in subjects with primary HTG is low. The low frequency of rare mutations, the absence of a more severe phenotype or the dominant transmission of the HTG would not suggest the use of genetic analysis in the clinical practice in this population.

**Electronic supplementary material:**

The online version of this article (doi:10.1186/s12944-016-0251-2) contains supplementary material, which is available to authorized users.

## Background

Hypertriglyceridemia (HTG) is a common complex metabolic trait resulting in increased plasma triglyceride (TG) levels, atherosclerosis risk, metabolic syndrome and in severe cases, high risk of acute pancreatitis [1]. HTG has been generally classified as primary, when a familial or inherited basis is suspected, whereas secondary HTG refers to cases in which coexist one or more identifiable conditions favouring HTG, including metabolic syndrome, type 2 diabetes, excess alcohol consumption, obesity, renal disease or certain drugs [2]. Selected primary very high HTG cases from lipid clinics, usually defined with TG > 500 mg/dL, are the result of the accumulation of relatively common genetic variants, with small to modest effect on TG, and/or the presence of rare mutations with large effect on TG in combination with other modifier genes and environmental factors [3, 4]. Hence, genetic studies in most HTG are predicted to add little information in their clinical management. In contrast, primary severe HTG with plasma triglyceride concentrations > 1000 mg/dL [5] concentrate rare mutations with major effects in genes involved in triglyceride-rich lipoprotein metabolism, including *LPL*, *APOC2*, *APOA5*, *LMF1, APOE* and *GPIHBP1* genes [6–9]. Homozygosity or compound heterozygosity for severe mutations in these genes comprise the most severe cases of HTG, with TG well over 1,000 mg/dL and recurrent episodes of pancreatitis since childhood in many occasions. However, these autosomal recessive defects are extremely rare, approximately 1:1,000,000, and most cases of very high HTG do not fulfil these criteria [10].

The prevalence of very high HTG is highly variable among populations, possibly caused by differences in environmental factors like diabetes prevalence or alcohol consumption, genetic background, and cohort selection. However, the prevalence of very high TG and the frequency of rare mutations causing HTG in a whole non-selected population have not been previously studied. This information could be very useful to establish the role of genetic testing and familial cascade screening for primary HTG.

## Methods

### Study subjects

The Spanish public health system distributes the population in different health districts, approximately 20,000–30,000 patients per district. Each health district includes 100 % of citizens living in that district. They share a single primary care facility, a single laboratory with computerized data since 2005, and a centralized registry of drugs. For this study, we selected all subjects of a primary care district (Centro Salud Almozara) in a middle class area of Zaragoza city in northern Spain. This facility is responsible of the primary health care of 23,310 subjects, which includes voluntary periodic examinations with fasting blood test every 5 years. All adult subjects (≥18 years of age) of Centro Salud Almozara were included in the study to establish the frequency of HTG in general population.

### HTG definition and HTG subjects selection

Very high HTG was defined with fasting TG > 500 mg/dL as proposed by Adult Treatment Panel III guideline recommendations [5]. HTG subject was defined as the presence in the last 10 years of very high TG, and at least two other measurements with TG >200 mg/dL. Subjects under treatment with drugs to decrease triglycerides (fibrates, omega-3 fatty acids or niacin) were considered as HTG in presence of a single TG value > 500 mg/dL. Secondary very high HTG was an exclusion criterion, and was considered in presence of uncontrolled diabetes (Hb1Ac >7.5 %) , obesity (body mass index >30 Kg/m2), alcohol abuse (>30 g/day for men and >20 g/day for women), renal disease (glomerular filtration rate <30 mL/min), liver disease (ALT >3 times upper normal limit) except non-alcoholic fatty liver, hypothyroidism (TSH >6 μUI/mL), pregnancy, hemochromatosis, autoimmune diseases, and concomitant use of estrogens (except contraceptive pills), any dose of oral corticosteroids or protease inhibitors.

### Normolipemic subjects

The normolipemic group consisted of healthy, unrelated men and women volunteers from the same facility, matched by age with the HTG group. Exclusion criteria for control subjects were TG >100 mg/dL, diabetes, current or past history of lipid-lowering drug consumption and *APOE* genotype other than ε3/ε3.

All selected subjects, cases and controls, signed informed consent to a protocol previously approved by our local ethical committee (Comité Ético de Investigación Clínica de Aragón, Zaragoza, Spain).

### Clinical and laboratory determinations

All participants were assessed for personal and familial history of cardiovascular disease, history of pancreatitis, medication use, cardiovascular risk factors and anthropometric measurements. Dietary intake in cases and controls was determined by interview with one single registered dietician. In this interview, a Spanish validated 137-item food frequency questionnaire (FFQ) was used [11].

EDTA plasma and serum samples were collected after 10–12 hours of fasting. Cholesterol and TG levels were determined by standard enzymatic methods. HDL cholesterol was measured directly by an enzymatic reaction using cholesterol oxidase (UniCel DxC 800; Beckman Coulter, Inc., Brea, CA, USA). Apo A1, apo B, lipoprotein(a) and C-reactive protein were determined by IMMAGE kinetic nephelometry (Beckman-Coulter, Inc.).

### Genetic analysis

DNA was extracted by standard methods. Promoters, coding regions and intron-exon boundaries of *LPL* (NM_000237), *LMF1* (NM_022773), *APOA5* (NM_052968), *APOC2* (NM_000483.4) and *GPIHBP1* (NM_178172) were amplified by PCR and purified by ExoSap-IT (USB) using primers shown in (Additional file 1: Table S1). Amplified fragments were sequenced by Sanger method using the BigDye 3.1 sequencing kit (Applied Biosystems) in an automated ABI 3500xL sequencer (Applied Biosystems). DNA sequences were analyzed using VariantReporter™ software (Applied Biosystems). *APOE* genotype was determined as previously described [12].

Rare and common variants were defined by frequencies <1 % and >1 % respectively of that obtained from the 1000 Genomes project [13]. Variants that were not previously described and absent in our control population were considered as rare. Rare and common variants were considered pathogenic if previously associated with HTG or considered pathogenic by the evaluation with predictive software in a bioinformatic analysis.

To evaluate the pathogenicity of newly identified genetic variants, we used PolyPhen-2 [14] and Mutation Taster [15]. The effect of variants in potential splicing sites was predicted with NetGene2 [16] and NNSplice [17]. We refer to non-synonymous variants as sequence variations causing amino acid substitutions (missense variants) or introducing of a premature stop codon (nonsense variants). Intronic variants were considered when they were located in intron-exon boundaries.

In order to compare the frequency of identified variants with that of the general population we have compiled the allele frequencies of the identified variants from the 1000 Genomes Project [13].

### Statistical analysis

Analyses were performed using SPSS version 20.0 (IBM). The nominal level for significance was *p* < 0.05. Normal distribution of variables was checked by Kolmogorov–Smirnov test. Quantitative variables with normal distribution were expressed as mean ± standard deviation and were analyzed by T-Student test. Variables with a skewed distribution were expressed as median and interquartile range and were analyzed by Mann–Whitney U test. Qualitative variables were expressed as percentage and were analyzed by Chi-square test.

## Results

**Fig. 1:**
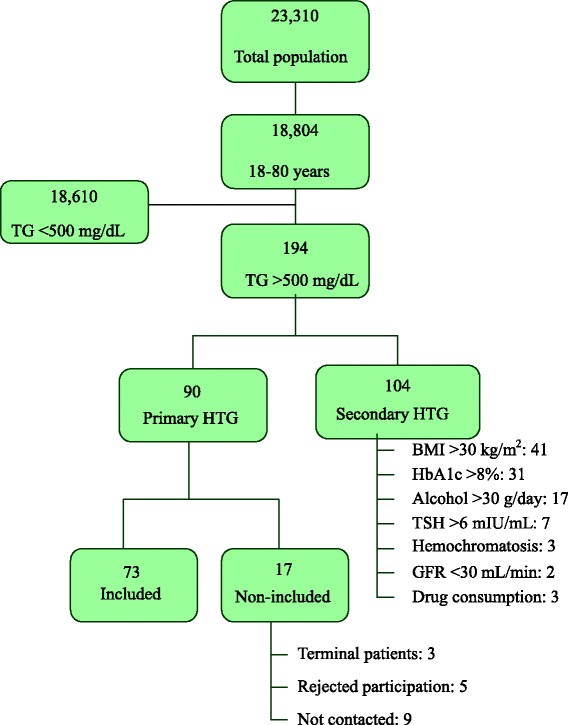
Flow chart of the population selected for this study

From the total selected population of 23310 subjects, 18804 subjects were aged 18–80 years, 194 (1.04 %) of them presented very high HTG in the last 10 years, and 90 subjects (46.4 %) met the inclusion criteria for primary HTG. The selection of study subjects group was carried out as indicated in the flow chart of Fig. 1. Finally, 73 patients with primary very high HTG were studied by sequencing the *LPL, LMF1, APOA5, APOC2, GPIHBP1* and *APOE* genes.

Anthropometric and clinical characteristics of the patients with primary HTG and control subjects are shown in Table 1. In the primary HTG group, there was a clear predominance of males (79.5 %) with a mean age of 56.4 years. Cardiovascular disease, diabetes and hypertension were more frequent among cases than in controls (Table 1).

**Table 1:** Clinical and biochemical characteristics of primary HTG and control groups^a^

	Primary HTG	Control	*p*
	*N* = 73	*N* = 90	
Age, years	56.4 ± 12.0	53.5 ± 16.2	0.210
Males, n (%)	58 (79.4)	45 (50.0)	0.000
Body mass index, kg/m^2^	27.6 ± 2.35	26.6 ± 3.25	0.044
Total cholesterol, mg/dL	264 ± 48.0	196 ± 34.1	0.000
Triglycerides, mg/dL	654 (550–810)	63.0 (49.7–74.0)	0.000
HDL cholesterol, mg/dL	40.1 ± 10.5	55.2 ± 12.6	0.000
Glucose, mg/dL	100 ± 16.1	91.3 ± 12.7	0.000
Hb1Ac (%)	5.6 (5.3–6.15)	5.3 (5.1–5.5)	0.000
Diabetes, n (%)	18 (24.7)	2 (2.3)	0.000
Hypertension, n (%)	31 (42.5)	13 (14.4)	0.000
Cardiovascular disease, n (%)	11 (15.1)	3 (3.3)	0.005
*APOE* genotype (%)			0.000
ε2- ε2	0	0	
ε2- ε3	19.7	0	
ε3- ε3	57.7	100	
ε3- ε4	16.9	0	
ε4- ε4	1.4	0	
ε4- ε2	4.2	0	

Quantitative variables are expressed as mean ± standard deviation, except for variables not following normal distribution, expressed as median (interquartile range). Qualitative variables are expressed as %. The p value was calculated by Student’s t test, Mann–Whitney U and Chi - square as appropriate.

^a^Lipid values refer to the most recent values without lipid-lowering treatment

Sequencing analysis of *LPL*, *LMF1*, *APOA5*, *GPIHBP, APOC2* and *APOE* genes revealed that nine patients out of 73 (12.3 %), were carriers of 7 rare variants: c.1018 + 1G > A in *LPL*, p.(Leu69Leu) and p.(Pro562Arg) in *LMF1,* p.(Leu173Pro) and p.(Gln97*) in *APOA5*, p.(Arg76Cys) in *GPIHBP1* and p.(Arg154Ser) in *APOE* . Three of them were described for the first time in this work: c.1018 + 1G > A in *LPL*, p.(Leu173Pro) in *APOA5*, and p.(Arg76Cys) in *GPIHBP1*. Only the variant p.(Pro562Arg) in *LMF1* was also present in controls (Table 2).

**Table 2:** Rare gene variants identified in the studied HTG population

Gene	Localization	Variant	Predicted aminoacid change	HTG group N (%)	Bioinformatic Analysis	Control group
*LPL*	Intron 6	c.1018 + 1G > A		2 (2.74 %)	Damage	0
*LMF1*	Exon 2	c.205	p.(Leu69Leu)	1 (1.37 %)	Damage	0
	Exon 11	c.1685C > G	p.(Pro562Arg)	1 (1.37 %)	Damage	2 (2.2 %)
*APOA5*	Exon 4	c.289C > T	p.(Gln97*)	1 (1.37 %)	Damage	0
		c.518T > C	p.(Leu173Pro)	1 (1.37 %)	Damage	0
*GPIHBP1*	Exon 2	c.226C > T	p.(Arg76Cys)	1 (1.37 %)	Damage	0
*APOE*	Exon 4	c.460C > A	p.(Arg154Ser)	2 (2.74 %)	Damage	0

Prediction of deleterious effects was performed using SIFT (http://sift.jcvi.org/), Polyphen-2 (http://genetics.bwh.harvard.edu/pph2/) and MutationTaster (http://www.mutationtaster.org)

For all the identified variants, the *in silico* analysis with MutationTaster and PolyPhen-2 was performed to evaluate the pathogenicity. The following variants were predicted as potentially harmful: c.-281G > C, c.1018 + 1 G > A, p.(Asp36Asn) and p.(Asn318Ser) in *LPL*; p.(Ser19Trp), p.(Gln97*), and p.(Leu173Pro) in *APOA5*, p.(Ser144Phe) and p.(Arg76Cys) in *GPIHBP1* and p.(Arg364Gln), p.(Pro562Arg), p.(Leu69Leu) in *LMF1*. This last variant in *LMF1*, although silent variant, was predicted as potentially harmful, since splicing score was increased (score wild type: 0.43 versus score mutant: 0.48).

Table 3 shows identified variants with allelic frequencies statistically different from those described in the 1000 Genomes project: p.(Asp36Asn) and c.-281T > G in *LPL*, and p.(Ser19Trp) and c.-3A > G in *APOA5*. Other polymorphisms previously associated with HTG [18, 19] were also present in our HTG population: p.(Ser474X) in *LPL*; and p.(Arg364Gln), p.(Arg354Trp) and p.(Pro562Arg) in *LMF1*, but their frequencies were not statistically different from those described in the 1000 Genomes project. The protector polymorphism, p.(Ser474X) in *LPL*, was found in our HTG population with lower frequency (11.1 %) that in the 1000 Genomes project (12.2 %), although this difference did not reach statistical significance.

**Table 3:** Comparison of allele frequencies of variants in our HTG population and 1000 Genomes project

Gene	Nucleotide change	Predicted aminoacid change	Frequency in HTG population	Frequency in the population 1000 Genomes	*p*	Bioinformatic Analysis
*LPL*	c.106G > A	p.(Asp36Asn)	0.055	0.013	0.001	Benign
	c.−281T > G		0.062	0.013	<0.001	Damage
*LMF1*	c.205C > T	p. (Leu69Leu)	0.007	0.0001	0.017	Damage
	c.288 + 298C > T		0.007	0.394	<0.001	Benign
*APOA5*	C.−3 A > G		0.185	0.080	<0.001	Benign
	c.56C > T	p.(Ser19Trp)	0.185	0.057	<0.001	Damage
	c.132C > A	p.(Ile44Ile)	0.178	0.056	<0.001	Benign
	c.162-43A > G		0.134	0.080	0.040	Benign
*GPIHBP1*	c.−509 G > A		0.164	0.442	<0.001	Unknown
	c. −208 G > C		0.240	0.443	<0.001	Unknown
	c.138G > T	p.(Val46Val)	0.281	0.443	0.0003	Benign
	c.295 + 19G > A		0.007	0.000	0.0226	Benign
	c.295 + 28G > A		0.007	0.000	0.0226	Benign
APOC2	c.−116T > A		0.50	0.376	0.004	Benign
	c.−89C > G		0.027	0.009	0.048	Benign
	c.56–30G > A		0.007	0.000	0.008	Benign
	c.216–81T > C		0.344	0.491	0.007	Benign

All variants are described in accordance with the latest recommendations of HGVS (http://www.hgvs.org/mutnomen). The *p* value was calculated by χ2 test

Prediction of deleterious effects was performed using SIFT (http://sift.jcvi.org/ ), Polyphen-2 (http://genetics.bwh.harvard.edu/pph2/) and MutationTaster (http://www.mutationtaster.org)

The clinical characteristics of the 9 patients carrying the 7 rare variants are shown in Table 4. The anthropometric and clinical data did not significantly differ between carriers and non-carriers of rare functional variants, including family history of HTG (Table 5).

**Table 4:** Clinical characteristics of patients carrying rare variants in *LPL, LMF1, APOA5, APOE* and *GPIHBP1* genes

Gene	Nucleotide change	Predicted aminoacid change	Age (years)	Sex	BMI (kg/m^2^)	TC, mg/dL	TG mg/dL	*APOE* Genotype
*LPL*	c.1018 + 1G > A		74	F	26.3	247	505	ε2/ε3
			59	M	29.7	374	1736	ε3/ε4
*LMF1*	c.205C > T	p. (Leu69Leu)	65	M	24.2	257	654	ε3/ε4
	c.1685C > G	p.(Pro562Arg)	28	M	26.85	286	932	ε2/ε3
*APOE*	c.460C > A	p.(Arg154Ser)	47	M	24.45	354	572	ε3/ε3
			64	M	29.1	287	586	ε3/ε3
*APOA5*	c.289C > T	p.(Gln97*)	53	M	23.9	252	1215	ε2/ε3
	c.518T > C	p.(Leu173Pro)	43	M	28.9	269	1410	ε3/ε4
*GPIHBP1*	c.226C > T	p.(Arg76Cys)	57	M	29.8	251	781	ε2/ε3

*BMI* Body Mass Index, *TC* Total Cholesterol, *TG* Triglycerides, *F* female, *M* male

**Table 5:** Clinical characteristics of HTG subjects according to presence/absence of rare variants

Clinical characteristics	Mutation carriers (*n* = 9)	Non-mutation carriers (*n* = 64)	*p*
Age (years)	59.8 ± 11	55,9 ± 12,2	0.017
Male n, (%)	8 (88.9 %)	50 (78.1 %)	0.058
BMI (kg/m^2^)	26.3 (24.0–29.4)	28,0 (25.9–29.7)	0.276
*APOE* genotype (%)			0.336
*ε2-ε3*	33.3	17.2	
*ε3-ε3*	33.3	62.5	
*ε3-ε4*	33.3	14.1	
*ε4-ε4*	0	1.6	
*ε4-ε2*	0	4.7	
Triglycerides, mg/dL	583 (529–1095)	669 (580–848)	0.416
Total cholesterol, mg/dL	281 ± 48.9	262 ± 48.1	0.743
HDL cholesterol, mg/dL	40.4 ± 9.67	40.1 ± 10.6	0.881
Diabetes, %	11.1	26.6	0.314
Hypertension, %	22.2	43.7	0.219
Cardiovascular disease, %	22.2	14	0.522
Familial history (%)	22.2	12.5	0.421

Quantitative variables are expressed as mean ± standard deviation, except for variables without normal distribution (median, interquartile range). The qualitative variables are expressed as %. BMI: body mass index

*P* value was calculated by Student’s t test, Mann - Whitney U and Chi – square, as appropriate

## Discussion

The prevalence of very high HTG in subjects aged 18 to 80 from the general population was 1.04 % in our study. To our knowledge, there are not similar studies including all (>99 %) subjects of a large community followed for 10 years. Due to the large variability of TG levels, we have considered those subjects with at least two separated determinations to confirm the high TG levels. The specific design of our study allows us to be confident about the consistency of our results. The characteristic of our national health system and the highly stable population, without significant immigration or emigration processes in the area of study in the last 10 years, reinforce our calculations. Furthermore, our results are concordant with other transversal studies such as the NHANES in a representative USA population, with an overall prevalence of 1.1 % of subjects with TG > 500 mg/dL [18]. Moreover, the frequency of HTG was 1.12 %, when defined as TG >400 mg/dL, in stratified sampling with a transversal design in the Spanish population [19]. This prevalence seems to be higher in other populations, especially Mexican American middle-aged men, probably due to differences in genetic background, diet, and prevalence of obesity [18].

Primary HTG is defined as a lipoprotein metabolism disturbance caused by alteration of genes and proteins that regulate lipid metabolism. Of the 194 subjects who had TG > 500 mg/dL, 104 met criteria for secondary HTG. This means that less than half of the HTG subjects (43.6 %) considered to have a predominantly genetic cause in our population, and is consistent with previous reports [20]. In our population, the major risk factor for secondary HTG was obesity (41.1 %). This percentage is similar to that found in the American population, in which 42.9 % of subjects with TG 200 mg/dL were classified as obese [18].

According to our results, the prevalence of HTG, both primary and secondary, was considerably lower among women than in men. In our HTG population, there was a clear predominance of males (79.5 %), as in the NHANES study, in which the 75.3 % of HTG were men [21], and also largely corroborated in other studies [22, 23]. The mechanism of this difference is not fully established. No locus related to triglycerides in sexual chromosomes has been found in GWAS, so the differences are probably due to different interaction of common gene variations with certain environmental factors. For example, a significantly greater efficiency of skeletal muscle to the postprandial clearance of both total and meal- derived triglycerides in women relative to men has been described and could explain a different impact of obesity on TG between genders [24].

Another objective our study was to determine the frequency of pathogenic mutations causing high TG levels in *LPL*, *LMF1*, *APOA5*, *GPIHBP1, APOC2* and *APOE* genes in subjects with primary HTG. The analysis of the pathogenicity of the identified variants revealed that 9 out of 73 subjects were carriers of 7 disease causing variants in the candidate genes. Therefore, 12.3 % of our population had a disease causing variant. This frequency is similar to that observed by Wang et al., who studied 110 subjects with severe HTG and analyzing rare variants in *LPL*, *APOA5* and *APOC2* genes obtained a mutation frequency of 10.9 % [22].

Of the 7 variants disease causing, only one of them, p.(Pro562Arg) in *LMF1,* is present in controls. This mutation has been associated with HTG in previous studies [4–25] and its frequency in the general population of the 1000 Genomes project is lower than 1 % [13]. However, it would be necessary to perform further functional analysis to analyze the potencial effect of this variant.

The silent variant p.(Leu69Leu) in *LMF1* was found to be pathogenic according to bioinformatic analysis. It does not generate an amino acid change, but it could generate a change in the physiological splicing according to bioinformatic analysis. Silent variants have been described as pathogenic by previous studies, showing that these variants can influence binding of regulatory factors on DNA, mRNA secondary structure and stability, ribosome traffic on mRNA and its interactions with specific ligands (as in riboswitches), including other RNAs and proteins [26]. It would be necessary to perform a functional analysis to study the potential effect of this variant.

Among the mutations identified in our population, three of them have been described for the first time in this work: p.(Leu173Pro), located in *APOA5* exon 4, that generates an amino acid change predicted as harmful by bioinformatic analysis; c.1018 + 1 G > A, that causes a change in splicing site in *LPL* intron 6, and p.(Arg76Cys), located in *GPIHBP1* exon 2 and producing an amino acid change also predicted as harmful by *in silico* analysis. Among our cases, none homozygous or compound heterozygous subject was found. Taking into account that these mutations were not found in the normolipemic population, we considered that these variants are playing an important role in the HTG phenotype of carrier subjects, and that severe HTG could be considered a co-dominant disease with variable penetrance. There is a large heterogeneity in the variants identified in our study. Only the changes c.1018 + 1G > A in *LPL* and p.(Arg154Ser) in *APOE* were present in two unrelated affected subjects. This is in agreement with observations in other genetic diseases in our population and could be due to the absence of a common genetic background in Spain [27].

One important issue in our results is the lack of clinical differences between cases with and without mutation. Therefore, the presence of a rare pathogenic mutation did not confer a differential phenotype or risk with respect to those subjects without mutations, neither useful information in the management of these subjects.

## Conclusions

In conclusion, we are far from knowing the full genetic basis of severe HTG, but the presence of rare mutations in candidate genes do not appear to be responsible in most cases. The low frequency of rare mutations in candidate genes and the absence of a more severe phenotype and family history of dominant transmission in those with rare mutations would not suggest the use of genetic analysis in clinical practice in this type of HTG. It would be possible that the study of known genes with minor effects on triglycerides like *GPD1, GCKR, CREB3L3, MET or INSR* the discovery of new HTG genes, or the use of new large scale tecniques may change this approach in the future [28, 29].

## Additional files

Additional file 1:
**Tables S1.** Primers used for sequencing of candidate genes. (DOCX 109 kb)

## Abbreviations

FFQ: food frequency questionnaire
HTG: hypertriglyceridemia
TG: triglyceride
