# Supplementary material for: Frequency of rare mutations and common genetic variations in severe hypertriglyceridemia in the general population of Spain
Source: Lipids Health Dis. 2016 Apr 23;15:82. doi: 10.1186/s12944-016-0251-2 (PMC4842266; doi:10.1186/s12944-016-0251-2)
Supplement: Additional file 1: — Tables S1. Primers used for sequencing of candidate genes. (DOCX 109 kb) [file 12944_2016_251_MOESM1_ESM.docx]

**Supplementary Table 1** Primers used for sequencing of candidate genes

| Gene | Primer | | | Product |
| --- | --- | --- | --- | --- |
|  | Exon | Name | Sequencing |  |
| *LPL* | 1^U^ | Forward  Reverse | 5´- TGTAAAACGACGGCCAGTAGCGAACAGGAGCCTAACAAAGCAA- 3´  5´- CAGGAAACAGCTATGACCTTTGGCGCTGAGCAAGTCGC-3´ | 340 |
|  | 1^D^ | Forward  Reverse | 5´- TGTAAAACGACGGCCAGTCACTTCTAGCTGCCCTGCCA-3  5´- CAGGAAACAGCTATGACCAGGGGAGTTTGCGCGCAAAA-3 | 330 |
|  | 2 | Forward  Reverse | 5´- TGTAAAACGACGGCCAGTAACCCTCCAGTTAACCTCATATCCAA-3  5´- CAGGAAACAGCTATGACCCACCACCCCAATCCACTCTTCCCAC-3´ | 227 |
|  |  |  |  |  |
|  | 3 | Forward  Reverse | 5´-TGTAAAACGACGGCCAGTTAGGTGGGGTATTTTAAGAAAGCTGTG-3´  5´-CAGGAAACAGCTATGACCCACTGTTTTGGACACATAAGTCTCC-3 | 296 |
|  |  |  |  |  |
|  | 4 | Forward  Reverse | 5´-TGTAAAACGACGGCCAGTGCAGAACTGTAAGCACCTTCATTTC-3´  5´-CAGGAAACAGCTATGACCTTCACCTCTTATGATAAGACCAACGAA-3´ | 180 |
|  |  |  |  |  |
|  | 5 | Forward  Reverse | 5´-TGTAAAACGACGGCCAGTAAATTTACAATCTGTGTTCCTGCTTTTT-3  5´-CAGGAAACAGCTATGACCGATAAGAGTCACATTTAATTCGCTTCTA-3 | 350 |
|  |  |  |  |  |
|  | 6 | Forward  Reverse | 5´- TGTAAAACGACGGCCAGTTTCTGCCGAGATACAATCTTGGTGTC-3´  5´-CAGGAAACAGCTATGACCGACTCCTTGGTTTCCTTATTTACAACA-3´ | 359 |
|  |  |  |  |  |
|  | 7 | Forward  Reverse | 5´-TGTAAAACGACGGCCAGTATAAAGATTGATCAACATGTTCGAATTTC-3  5´-CAGGAAACAGCTATGACCGGGACTGGTGCCATGATGACCGCCC-3´ | 237 |
|  |  |  |  |  |
|  | 8 | Forward  Reverse | 5´-TGTAAAACGACGGCCAGTGATCTCTATAACTAACCAATTTATTGCT-3  5´-CAGGAAACAGCTATGACCTGGGGGTCTAAAGTGAAGGAAGAAAA-3´ | 299 |
|  |  |  |  |  |
|  | 9 | Forward  Reverse | 5´-TGTAAAACGACGGCCAGTTTGTTCTACATGGCATCTTCACATCCA-3´  5´-CAGGAAACAGCTATGACCAGCTCAGGATGCCCAGTCAGCTTTA-3´ | 310 |
|  |  |  |  |  |
| *LMF1* | 1 | Forward  Reverse | 5´-GTGCCTCCGGGACTGTGA-3  5´- CGGAGGAGTCTCGAGGGA-3´ | 422 |
|  |  |  |  |  |
|  | 2 | Forward  Reverse | 5´- ATTCTGAGCTGCGCCCAT-3´  5´- CAGTGCCTGTGCTGAGTGAC -3´ | 466 |
|  |  |  |  |  |
|  | 3 | Forward  Reverse | 5´- CAAGCCAAAGTGTTAATACTCGTTTC-3´  5´- GAAGGCTGATGGCAGAGGCTAAGGAA-3´ | 170 |
|  |  |  |  |  |
|  | 4 | Forward  Reverse | 5´- GGCTGGTGTCTCTCAGTAGCA-3´  5´- AAGCCCTCACAGGTTAGAAGAG- 3´ | 345 |
|  |  |  |  |  |
|  | 5 | Forward  Reverse | 5´- CTTCGTGGATGGTTCGTCTT-3´  5´- TGATGCGACAGCTCACCA-3´ | 269 |
|  |  |  |  |  |
|  | 6 | Forward  Reverse | 5´- GGGGATCCTGTGTGCAGTAG-3´  5´- GGGCAGCCAGAAATAGGG-3´ | 354 |
|  |  |  |  |  |
|  | 7 | Forward  Reverse | 5´- GCTCCAGGAAGAGAGGCG-3´  5´- CCGACTTTCTCCTGCCCT-3´ | 308 |
|  |  |  |  |  |
|  | 8 | Forward  Reverse | 5´- AGCAGCAGCTGGGGTCTC-3´  5´- GCACTGTAACCCCACCTGAA-3´ | 320 |
|  |  |  |  |  |
|  | 9 | Forward  Reverse | 5´- ATGGACAGTCGGGGAACC-3´  5´- AAGAGGGTGGGGGTACAG-3´ | 330 |
|  |  |  |  |  |
|  | 10 | Forward  Reverse | 5´- ATGGACAGTCGGGGAACC-3´  5´- AAGAGGGTGGGGGTAC-3´ | 259 |
|  |  |  |  |  |
|  | 11 | Forward  Reverse | 5´- CAGCAGCAGGCTGAGGAG-3´  5´- CTCTCCTCTCCACGTCTCTCTT-3´ | 363 |
|  |  |  |  |  |

| Gene | Primer | | | Product |
| --- | --- | --- | --- | --- |
|  | Exon | Name | Sequencing |  |
| *APOA5* | 1 | Forward  Reverse | 5´- GTGAGTGCTGGGAGGCAGCTGAGGTCAACTT-3´  5´- CCACCTGCAAT//GCCCTCCCTTAGGACTGTG-3´ | 184 |
|  | 2-3 | Forward  Reverse | 5´- GCATTGCAGGTGG//CGCCATGTCCCTTC-3´  5´- ATGGCCCAGCTGTGTCCTCCCTTCGCCTACA-3´ | 408 |
|  | 4.1 | Forward  Reverse | 5´- CAGAGGATCAGTGCGCGATGACTTG-3´  5´- ATGGCGCGAGTGAAGGCAGCTATC-3´ | 834 |
|  | 4.2 | Forward  Reverse | 5´- GAGGTGCGCCAGCGACTT-3´  5´- GGCGTGCTCTTGCTACCTC-3´ | 1042 |
| *APOC2* | 1 | Forward  Reverse | 5´- GCTGTGTCCAAGTCCATGC- 3´  5´- GGGGGAGAGTGTGTCAGGAG-3´ | 379 |
|  | 2-3 | Forward  Reverse | 5´- CTGCCCTCTCCTCTTCTTCC-3´  5´- TCTGGGTCCTGGATGCAGT -3´ | 302 |
|  | 4 | Forward  Reverse | 5´- CATACCTGCCCGCTGTAGAT-3´  5´- TCAGGCTAGAGTTGGGAGGA-3´ | 538 |
| *GPIHBP1* | 1 | Forward  Reverse | 5´- CAAAGACCCGAGGTGATAGTG-3´  5´- CCCCTCCTTCTTCCTAAGCC-3´ | 770 |
|  | 2 | Forward  Reverse | 5´- GGAGTTGGGGGCACGATG-3´  5´- TGAGCAGCAGTGAGGGGTGT-3´ | 365 |
|  | 3 | Forward  Reverse | 5´- GCTCACCAGGCTAGGCTTTG-3´  5´- TTTGCCAGGGTGGGACAT-3´ | 487 |
|  | 4 | Forward  Reverse | 5´- TCCTGACCACCCACTCCACG-3´  5´- CGCCCCTCCGCATGTG-3´ | 834 |
